# Molecular epidemiology of carbapenem-resistant *Enterobacterales* in Thailand, 2016–2018

**DOI:** 10.1186/s13756-021-00950-7

**Published:** 2021-06-05

**Authors:** Wantana Paveenkittiporn, Meghan Lyman, Caitlin Biedron, Nora Chea, Charatdao Bunthi, Amy Kolwaite, Noppavan Janejai

**Affiliations:** 1 National Institute of Health, Department of Medical Sciences, Ministry of Public Health, Nonthaburi, Thailand; 2 National Center for Emerging and Zoonotic Infectious Diseases, Centers for Disease Control and Prevention, 1600 Clifton Road, MS H16-3, Atlanta, GA 30329-4027 USA; 3 Center for Surveillance, Epidemiology, and Laboratory, Centers for Disease Control and Prevention, Atlanta, GA USA; 4 Division of Global Health Protection, Center for Global Health, Centers for Disease Control and Prevention, Atlanta, GA USA

## Abstract

**Background:**

Carbapenem-resistant *Enterobacterales* (CRE) is a global threat. *Enterobacterales* develops carbapenem resistance through several mechanisms, including the production of carbapenemases. We aim to describe the prevalence of Carbapenem-resistant *Enterobacterales* (CRE) with and without carbapenemase production and distribution of carbapenemase-producing (CP) genes in Thailand using 2016–2018 data from a national antimicrobial resistance surveillance system developed by the Thailand National Institute of Health (NIH).

**Methods:**

CRE was defined as any *Enterobacterales* resistant to ertapenem, imipenem, or meropenem. Starting in 2016, 25 tertiary care hospitals from the five regions of Thailand submitted the first CRE isolate from each specimen type and patient admission to Thailand NIH, accompanied by a case report form with patient information. NIH performed confirmatory identification and antimicrobial susceptibility testing and performed multiplex polymerase chain reaction testing to detect CP-genes. Using 2016–2018 data, we calculated proportions of CP-CRE, stratified by specimen type, organism, and CP-gene using SAS 9.4.

**Results:**

Overall, 4,296 presumed CRE isolates were submitted to Thailand NIH; 3,946 (93%) were confirmed CRE. Urine (n = 1622, 41%) and sputum (n = 1380, 35%) were the most common specimen types, while blood only accounted for 323 (8%) CRE isolates. The most common organism was *Klebsiella pneumoniae* (n = 2660, 72%), followed by *Escherichia coli* (n = 799, 22%). The proportion of CP-CRE was high for all organism types (range: 85–98%). Of all CRE isolates, 2909 (80%) had one CP-gene and 629 (17%) had > 1 CP-gene. New Delhi metallo-beta-lactamase (NDM) was the most common CP-gene, present in 2392 (65%) CRE isolates. *K. pneumoniae* carbapenemase (KPC) and Verona integron-encoded metallo-β-lactamase (VIM) genes were not detected among any isolates.

**Conclusion:**

CP genes were found in a high proportion (97%) of CRE isolates from hospitals across Thailand. The prevalence of NDM and OXA-48-like genes in Thailand is consistent with pattern seen in Southeast Asia, but different from that in the United States and other regions. As carbapenemase testing is not routinely performed in Thailand, hospital staff should consider treating all patients with CRE with enhanced infection control measures; in line with CDC recommendation for enhanced infection control measures for CP-CRE because of their high propensity to spread.

**Supplementary Information:**

The online version contains supplementary material available at 10.1186/s13756-021-00950-7.

## Introduction

Antimicrobial resistance (AMR) is a global health priority considering its association with high morbidity and mortality and increased healthcare costs [1–3]. The burden of AMR is growing worldwide with transmission often occurring in healthcare settings due to poor infection control practices and inappropriate use of antimicrobials. The threat of AMR is particularly concerning in low- and middle-income countries such as Thailand where AMR contributes to approximately 38,000 deaths per year and additional US$1.2 billion in healthcare costs [4]. Thailand’s AMR burden has increased over the past two decades, likely a result of excessive and inappropriate use of antimicrobials and poor sanitation [5–7].

Carbapenem-resistant *Enterobacterales* (CRE) are an especially concerning AMR threat because they are resistant to many last-resort antibiotics, making it difficult to treat and leading to high mortality rates. CRE are often the result of healthcare transmission and associated with risk factors such as previous antibiotic use, prolonged hospitalization, and medical device use [8–11]. CRE develops antibiotic resistance through several mechanisms, including through the production of carbapenemases, enzymes that degrade carbapenem antibiotics. Evidence suggests distinct differences in the epidemiology of CRE with and without carbapenemase production. Carbapenemase producing CRE (CP-CRE) are more virulent and are associated with higher levels of antimicrobial resistance, worse outcomes, and more rapid spread, while noncarbapenemase-producing CRE (non-CP-CRE) have been associated with asymptomatic carriage and perhaps less person-to-person transmission [12–14]. For this reason, CP-CRE have been identified as an important target for prevention sometimes warranting enhanced infection control interventions [15].

Carbapenemase enzymes are encoded by genes on mobile genetic elements, such as plasmids, which are highly transmissible between organisms and increase the potential spread of resistance. There are several genes encoding different carbapenemases, including *Klebsiella pneumoniae* carbapenemase (KPC) which is the most common carbapenemase in the United States and New Delhi metallo-beta-lactamase (NDM) which was first identified in a traveler returning from India but has now spread worldwide. Others include Verona integrin-encoded metallo-β-lactamase (VIM) and imipenemase (IMP) which are most prevalent in Southern Europe and Asia and oxacillinase-48 (OXA-48) which is most prevalent in the Mediterranean region, Europe, and India [16].

The National Antimicrobial Resistance Surveillance center, Thailand (NARST) which is managed by Thailand’s National Institute of Health (NIH), was established in 1998 and includes 92 hospital laboratories from all five regions of Thailand. NARST data show the overall percentage of *K. pneumoniae* isolates resistant to meropenem increased from 0.4% in 2000 to 6.5% in 2016 and the overall percentage of *Escherichia coli* isolates resistant to meropenem increased from 0.6 to 1.6% during the same period [6]. In August 2016, with growing concerns about AMR and CRE burden and antimicrobial effectiveness, the Ministry of Public Health of Thailand endorsed a National Strategic Plan on AMR to reduce the mortality, morbidity, and economic impact of AMR.

NARST contains limited information on resistance genes because resistance mechanism testing is not routinely performed at participating hospital laboratories. NIH established the Enhanced Incorporation of Global and National AMR (EIGNA) Surveillance System to understand the distribution of resistance genes and identify novel resistance genes among CRE in Thailand. We analyzed 2016–2018 EIGNA data to better understand the distribution of resistance genes and identify novel resistance genes among CP-CRE in Thailand.

## Methods

EIGNA includes 25 of 92 hospital laboratories participating in NARST surveillance from all five regions of Thailand; 21 (84%) of the labs are regional, tertiary care hospitals (Table 3). These laboratories were chosen based on their history of timely and complete submission of NARST data, the presence of competent laboratory performance based on external quality assessments and site visits, and a functional infection prevention and control (IPC) program.

Hospital laboratories performed Gram stains, cultures, biochemical tests, and antimicrobial susceptibility testing (AST) using disk diffusion and/or E-test on routine clinical specimens from hospitalized patients. CRE was defined as any *Enterobacterales* isolate resistant to ertapenem, imipenem, or meropenem according to CLSI M100-S26 standards [17]. Participating hospitals were instructed to submit the first CRE isolate from each specimen type and each patient admission to the NIH along with a case report form (CRF) containing patient demographics, admission date, discharge date, previous antibiotic exposure, patient comorbidities, and outcome of hospitalization.

NIH repeated AST using disk diffusion. E-test was done to confirm CRE for isolates that had discordant results. CRE isolates confirmed by NIH underwent multiplex polymerase chain reaction (PCR) to detect five carbapenemase-producing (CP) genes, including *bla*KPC, *bla*NDM, *bla*OXA-48-like, *bla*VIM, and *bla*IMP [18]. As colistin is a drug of last resort for serious infections due to multidrug resistant organisms, PCR testing was also done to detect *bla*MCR-1 (i.e., plasmid-mediated mobilized colistin resistance). Since hyperproduction of AmpC β-lactamase combined with altered membrane permeability can result in carbapenem resistance [12, 19, 20], routine PCR testing for *bla*AmpC was started for CRE isolates in the middle of this surveillance period and included testing for some isolates from 2017 and all isolates from 2018. Isolates negative by PCR for the six targeted genes were tested using modified Carbapenem Inactivation Method (mCIM) [21] for carbapenemase production and with commercial AMR Direct Flow Chip Kit (Master Diagnostica, Seville, Spain) [22] to confirm the absence of targeted CP genes and the presence of any additional CP genes.

For isolates with evidence of carbapenemase production but no CP gene identified, whole-genome sequencing was conducted by isolating DNA from overnight cultures with a DNeasy Blood and Tissue kit (Qiagen, Hilden, Germany) and quantifying the extracted DNA using the Qubit dsDNA HS Assay Kit (Invitrogen), both according to the manufacturer’s protocols. Genomic libraries were generated with the QIAGEN® QIAseq FX DNA Library Kit (Qiagen, Hilden, Germany) following to the manufacturer’s protocol. The Whole-genome sequencing (WGS) was carried out using the Illumina® MiSeq platforms to obtain 250-bp paired-end reads chemistry (Illumina, California, United States) according to manufacturer’s instructions. The 250-bp paired-end reads was de novo assembled using the CLC Genomics Workbench 12.0.2 (Qiagen, Aarhus, Denmark) using defaults settings except that the minimum contig size threshold was set to 500 bp in length.

EIGNA data from September 2016 through August 2018 were analyzed using SAS 9.4 (SAS Institute Inc., Cary, NC). Proportions of CP-CRE isolates among CRE isolates were calculated and stratified by specimen type, organism, and carbapenemase-producing gene. For the analysis of patient characteristics, isolate data was deduplicated by including CRF information from the first isolate submitted. However, for one patient whose first isolate was CP-CRE negative and subsequent isolates were CP-CRE positive, the first CP-CRE isolate was included and prior non-CP-CRE isolates were excluded. Clinical characteristics were calculated for CP-CRE and non-CP-CRE patients and compared using the Wilcoxon-Mann–Whitney test for continuous variables and chi-square and Fisher’s exact tests for categorical variables.

## Results

**Fig. 1:**
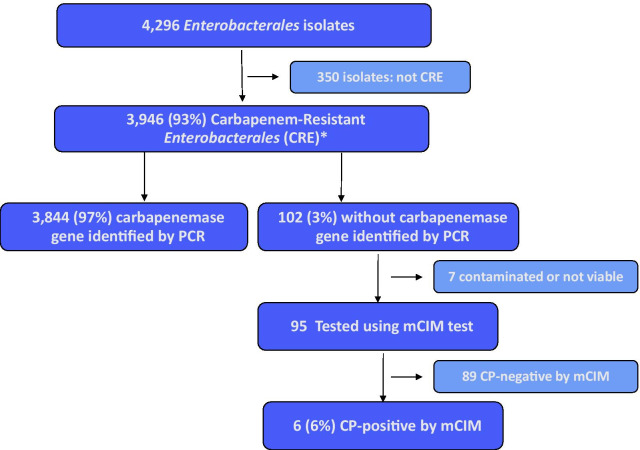
Testing results of *Enterobacterales* surveillance isolates submitted, Thailand, 2016–2018. *1 additional CRE isolate with no genotype results available was not included in the analysis

A total of 4296 presumed CRE isolates were submitted to NIH by participating hospital laboratories and 3946 (92%) isolates from 3748 patients were confirmed by NIH as CRE [Fig. 1], with 224 patients having multiple CRE isolates of different specimen types or pathogens. Urine and sputum were the most common specimen types (41% from urine, 35% from sputum), while blood accounted for only 8% of all CRE specimens. *K. pneumoniae* (73%) and *E. coli* (21%) were the most common organisms among all confirmed CRE isolates [Table 1].

**Table 1:** Frequency of Carbapenem-resistant *Enterobacterales (CRE)* and Carbapenemase-producing (CP)-CRE isolates by specimen type and organism, Thailand, 2016–2018

Isolate characteristics	No. CRE isolates (%)	No. CP-CRE isolates (%)	% of CRE with CP gene
Specimen type			
Urine	1622 (41%)	1567 (41%)	97
Sputum	1380 (35%)	1351 (35%)	98
Pus	437 (11%)	429 (11%)	98
Blood	323 (8%)	320 (8%)	99
Other normally sterile site	77 (2%)	75 (2%)	97
Other	107 (3%)	102 (3%)	95
Organism			
*Klebsiella pneumoniae*	2888 (73%)	2814 (73%)	97
*Escherichia coli*	825 (21%)	816 (21%)	99
*Enterobacter cloacae*	123 (3%)	115 (3%)	93
*Citrobacter freundii*	11 (< 1%)	11 (< 1%)	100
Other organism	99 (3%)	88 (2%)	89
**Total**	**3946**	**3844**	**97**

Of 3748 patients with confirmed CRE, 2368 (63%) were collected more than two calendar days after admission (suggesting healthcare onset [HO]), 2502 (67%) had antibiotic exposures during 14 days prior to specimen collection, and 885 (24%) died during hospitalization. For the 3724 (99%) with information on age available, median age was 66 years (IQR: 52–77). Of the 3031 (80%) with information on length of stay (LOS) available, median LOS was 20 days (IQR: 9–39). Of the 3318 (80%) with information on duration from admission to specimen collection date available, median duration was 10 days (IQR: 2–23) [Table 2].

**Table 2:** Characteristics of patients with and without a carbapenemase gene, Thailand, 2016–2018

Patient characteristic	n (% or IQR)			*p* value**
	All CRE	CP-CRE	Non-CP-CRE	
Median age (N = 3,724)	66 (52–77)	62 (52–77)	65 (45–79)	0.75
No. male (N = 3,748)	2263 (60)	2211 (61)	53 (54)	0.36
No. with any antibiotic 14 days prior (N = 3,748)	2502 (67)	2436 (67)	66 (67)	0.98
Median length of stay (LOS) (N = 3,031)	20 (9–39)	20 (9–39)	24 (11–38)	0.18
Median LOS before specimen collection*** (N = 3,318)	10 (2–23)	10 (1–23)	10 (3–24)	0.51
Median LOS after specimen collection *** (N = 2,968)	9 (4–19)	9 (4–19)	11 (4–19)	0.48
No. with healthcare onset (N = 3,748)	2368 (63)	2298 (63)	70 (71)	0.12
Death during hospitalization (N = 3,748)	885 (24)	864 (24)	21 (21)	0.57

^**^Wilcoxon-Mann–Whitney test used for numeric variables

^***^includes only those where LOS ≥ 0 (44 where LOS total < 0; 101 where LOS before specimen collection < 0; 107 where LOS after specimen collection < 0)

Of 3,946 CRE isolates, 3,844 (97%) tested positive for at least one CP gene and the proportion of CP-CRE for all *Enterobacterales* species ranged from 89 to 100% [Table 1]. These CP-CRE isolates were submitted from 25 hospitals across all five geographic regions of Thailand, with 21 of these hospitals (84%) having > 500 beds [Table 3], and the percentage of CRE isolates with a CP gene ranging from 94–100% across hospitals and 97–99% across all regions [Additional file 1: Table 1]. On univariate analysis, there were no statistically significant differences between CP-CRE and non-CP-CRE patients with regard to age, gender, use of any antibiotic during the two weeks prior to culture, HO status, total length of hospital stay, length of stay before or after specimen collection, or death during hospitalization [Table 2].

**Table 3:** Characteristics of participating facilities with Carbapenemase-producing (CP)-CRE isolates, Thailand, 2016–2018

Facility Characteristic	Total no. facilities (%) N = 25
Region	
North	6 (24%)
Northeast	6 (24%)
East	3 (12%)
Central	5 (20%)
South	5 (20%)
Bed size	
251–500	4 (16%)
501–750	13 (52%)
751–1000	7 (28%)
> 1000	1 (4%)

The distribution of carbapenemase genes among these isolates can be found in Table 4. Of all 3946 CRE isolates, 2501 (63%) were positive for *bla*NDM and 1892 (48%) were positive for *bla*OXA-48-like (48%). There were 634 (16%) isolates with coexistence of both *bla*NDM and *bla*OXA-48-like genes, seven (< 1%) isolates with coexistence of both *bla*IMP and *bla*OXA-48-like genes, and five (< 1%) isolates with coexistence of *bla*IMP and *bla*NDM. When stratified by organism, *bla*NDM remained the most common CP gene across all organisms, except in *K. pneumoniae*, which had a similar percentage of isolates with *bla*NDM (55%) and *bla*OXA-48-like (59%) genes. The highest proportion of *bla*NDM-positive isolates were among *Citrobacter freundii* (n = 11, 100%) and *E. coli* (n = 776, 94%). *E. cloacae* had the highest proportion of isolates positive for *bla*IMP (31, 25%). *bla*KPC and *bla*VIM genes were not detected among any of the isolates. Of the 102 isolates with no CP gene identified, the most common pathogen was *K. pneumoniae* (73, 72%). The distribution of CP genes by region is presented in Additional file 1: Table 1.Of the 102 isolates without a CP gene identified in the initial 5-target PCR, 95 isolates had a mCIM test done, six (6%) of which were mCIM positive, and 51 were tested for *bla*AmpC, four (8%) of which were positive. Of the six mCIM positive isolates, one was positive for both mCIM positive and *bla*AmpC and was later found to have *bla*IMI by whole-genome sequencing. One mCIM positive isolate was also later found to have Non-Metalloenzyme Carbapenemase (*bla*NMC) and *bla*IMI genes by AMR Direct Flow Chip kit. The MCR-1 gene was identified in ten isolates, including six isolates that also had *bla*NDM and three isolates that also had *bla*OXA-48-like.

**Table 4:** Presence of carbapenemase* and Mrc1 genes in isolates by organism, Thailand, 2016–2018

	n (% of isolates by organism)						*P* value^€^
Carbapenemase Gene	Total (N = 3946)	Organisms					
		*Klebsiella pneumoniae* (N = 2659)	*Escherichia coli* (N = 799)	*Enterobacter cloacae* (N = 113)	*Citrobacter freundii* (N = 12)	Other (N = 102)	
NDM	2501 (63)	1577 (55)	776 (94)	736 (59)	11 (100)	64 (65)	< .0001
OX-48-like	1892 (48)	1703 (59)	149 (18)	27 (22)	1 (9)	12 (12)	< .0001
IMP	97 (2)	46 (2)	1 (< 1)	31 (25)	0 (0)	19 (19)	< .0001
No CP Gene	102 (3)	74 (3)	9 (1)	18 (16)	0 (0)	1 (1)	< .0001
Mcr1	10 (< 1)	5 (< 1)	5 (1)	0 (0)	0 (0)	0 (0)	0.23

^*^Not mutually exclusive: 634 had NDM and OXA and 7 had IMP and OXA, 5 had NDM and IMP, 6 Mcr1 and NDM, 3 Mcr1 and OXA-48-like

^*^No VIM or KPC

€*p*-value using chi-square or fisher exact test to compare presence of carbapenemase genes and MCR-1 across organisms

## Discussion

The EIGNA surveillance system provides new information about carbapenemase distribution across Thailand. While previous studies show that there is a high burden of CRE in Thailand [6, 7, 23], our analysis demonstrates a high prevalence of CP genes among CRE in Thailand, with 97% of CRE isolates submitted to EIGNA having a CP gene. This estimate is much higher than estimates from other countries, such as the United States and India, where 32% and 28% of CRE are CP-CRE [24, 25]. This is also higher than recent estimates from a smaller study in Thailand which found that 71% of 287 *Enterobacterales* isolates from Bangkok were CP-CRE [26]. While these different estimates may reflect differences in the populations being studied, we found CP-CRE isolates from all regions of Thailand and the proportion of CRE with a CP gene was similar across regions. Despite some prior evidence of increased mortality associated with CP-CRE after adjusting for clinical factors [13], we did not identify a specific population at greater risk of CP-CRE based on demographics, prior antibiotic exposure, length of stay, or death, as there was no significant difference when comparing patients with CP-CRE and patients with non-CP-CRE. Yet, the low number of CP-CRE specimens from blood and sterile specimens reflects a low proportion of invasive infections with high mortality.

The distribution of CP genes in Thailand also differs from that found in other parts of the world. The most common CP genes in Thailand were NDM (69%) and OXA-48-like (38%), regardless of organism. These results support other studies showing that NDM is the most common gene in South and Southeast Asia [27–30]. However, this differs from the United States where NDM is present in 3% of isolates and OXA-48-like genes are present in 65% of isolates [25, 30]. KPC is the most common CP gene in the United States, present in 87% of CP-CRE isolates [25, 30], but was not present in any of these isolates from Thailand.

The U.S. Centers for Disease Control and Prevention recommends enhanced infection control measures specifically for CP-CRE compared to non-CP-CRE because of their high propensity to spread. However, testing for the presence of a CP gene is not routinely done in Thailand’s clinical laboratories and reference laboratory testing results are often delayed, hindering their use in guiding infection control measures. Given the high proportion of CP- CRE, clinicians in Thailand that do not have access to genotyping or carbapenemase production testing should consider that any CRE isolate is likely to have a CP gene and use appropriate infection control precautions for all patients with CRE. Testing for specific CP genes is more difficult and less available than testing for the presence of any carbapenemase activity. While there is currently no evidence or recommendation about special infection control measures associated with specific CP genes, information about CP genes can be useful to guide antimicrobial therapy. NDM is associated with limited treatment options. Newer combination drugs such as meropenem-vaborbactam and imipenem-relebactam are effective against KPC, but not NDM or OXA-48-like carbapenemases. Ceftazadime-avibactam is effective against both KPC and OXA-48-like carbapenemases, but not against NDM [31]. However the combination of ceftazidime-avibactam with aztreonam offers a therapeutic advantage against NDM, as well as being effective against KPC and OXA_48-like [32, 33]. The co-existence of multiple carbapenemase genes in the same isolate was frequent but the clinical implication is not well understood and should be further investigated to determine whether these patients have poorer outcomes or should be prioritized for enhanced infection control efforts.

## Limitations

There are several limitations to this study that restrict its generalizability. Participating sites were chosen based on microbiology capacity and surveillance performance to optimize data accuracy but may not reflect a nationally representative sample and gaps in reporting may have resulted in an underestimate of total CRE cases. Different hospital types and locations were included, but regional hospitals (82%), which provide tertiary care and likely have more severely ill patients, were disproportionately represented. Results may also have been biased by inconsistent culturing practices or laboratory testing at participating sites. For example, specimen collection and testing at hospital sites was left to the clinical discretion of each hospital or clinician and therefore not standardized. As a result, some clinicians may have collected samples only from patients with more severe conditions or those who did not respond to initial therapy with first-line antibiotics. Additionally, ertapenem susceptibility testing was not performed at all sites; only 14 (17%) of 84 sites participating in NARST do ertapenem susceptibility testing. As a result, isolates resistant to only ertapenem, which are more likely to be non-CP-CRE, may not have been identified or included in the analysis [34, 35]. Whole-genome sequencing was only conducted on a subset of isolates because of resource accessibility and may have limited detection of additional CP genes.

Clinical data collected as part of the EIGNA surveillance system was limited and did not include information about some risk factors or exposures (e.g., use of indwelling medical devices, prior hospitalizations or healthcare exposures, underlying comorbidities) which may be associated with CP-CRE. Outcome data was only reported for the hospitalization and did not include death after discharge.

## Conclusion

We found that carbapenemase production is predominant among CRE across Thailand. The distribution of carbapenemase-producing genes and the prevalence of NDM and OXA-48-like genes in Thailand differs from that in the United States and other regions of the world but seems to be consistent with patterns demonstrated by the limited data from Southeast Asia. Our analysis demonstrated a high frequency of established healthcare risk factors and poor outcomes among patients with CP-CRE. Efforts to strengthen clinical laboratory capacity for carbapenemase testing is important to improve early identification and appropriate response by guiding clinical treatment and control measures. Given that current availability of carbapenemase testing results is limited, all patients with CRE should be considered to have CP-CRE and treated with appropriate use of infection control measures. Ongoing CRE surveillance is necessary to detect new, emerging resistance mechanisms, monitor CRE trends, and determine the effectiveness of control measures in preventing transmission.

## Supplementary Information

**Additional file 1: Table 1**. Presence of carbapenemase* and MCR-1 genes in isolates by region, Thailand, 2016–2018.

## Data Availability

The datasets used and/or analyzed during the current study are available from the corresponding author on reasonable request.
