# Supplementary material for: Molecular epidemiology of carbapenem-resistant Enterobacterales in Thailand, 2016–2018
Source: Antimicrob Resist Infect Control. 2021 Jun 5;10:88. doi: 10.1186/s13756-021-00950-7 (PMC8180034; doi:10.1186/s13756-021-00950-7)
Supplement: Supplementary file 1 — Additional file 1: Table 1. Presence of carbapenemase* and MCR-1 genes in isolates by region, Thailand, 2016–2018. [file 13756_2021_950_MOESM1_ESM.docx]

Supplemental Table 1: Presence of carbapenemase* and Mrc1 genes in isolates by region, Thailand, 2016–2018.

|  | n (% of isolates by organism) | | | | | |
| --- | --- | --- | --- | --- | --- | --- |
| Carbapenemase Gene | Total  (N=3,946) | Organisms | | | | |
|  |  | Central  (N=1,060) | East  (N=381) | North  (N=895) | Northeast  (N=873) | South  (N=737) |
| NDM | 2,501 (63) | 610 (58) | 254 (67) | 676 (75) | 445 (51) | 516 (70) |
| OX-48-like | 1,892 (48) | 597 (56) | 141 (37) | 271 (30) | 516 (59) | 367 (50) |
| IMP | 97 (2) | 14 (1) | 35 (9) | 35 (4) | 1 (<1) | 12 (2) |
| No CP Gene | 102 (3) | 38 (4) | 17 (4) | 25 (3) | 11 (1) | 11 (1) |
| Mcr1 | 10 (<1) | 1 (<1) | 1 (<1) | 4 (<1) | 1 (<1) | 1 (<1) |

*Not mutually exclusive: 634 had NDM and OXA and 7 had IMP and OXA, 5 had NDM and IMP, 6 Mcr1 and NDM, 3 Mcr1 and OXA-48-like

*No VIM or KPC
